# Modeling noncontact atomic force microscopy resolution on corrugated surfaces

**DOI:** 10.3762/bjnano.3.26

**Published:** 2012-03-13

**Authors:** Kristen M Burson, Mahito Yamamoto, William G Cullen

**Affiliations:** 1 Materials Research Science and Engineering Center, Department of Physics, University of Maryland, College Park, Maryland 20742-4111, USA; 2 Center for Nanophysics and Advanced Materials, Department of Physics, University of Maryland, USA

**Keywords:** graphene, model, noncontact atomic force microscopy, SiO_2_, van der Waals

## Abstract

Key developments in NC-AFM have generally involved atomically flat crystalline surfaces. However, many surfaces of technological interest are not atomically flat. We discuss the experimental difficulties in obtaining high-resolution images of rough surfaces, with amorphous SiO_2_ as a specific case. We develop a quasi-1-D minimal model for noncontact atomic force microscopy, based on van der Waals interactions between a spherical tip and the surface, explicitly accounting for the corrugated substrate (modeled as a sinusoid). The model results show an attenuation of the topographic contours by ~30% for tip distances within 5 Å of the surface. Results also indicate a deviation from the Hamaker force law for a sphere interacting with a flat surface.

## Introduction

Noncontact atomic force microscopy (NC-AFM) has brought considerable advancement to the atomic-scale study of surfaces, by allowing both atomic-resolution imaging and atomically resolved force spectroscopy. Generally, these advancements have been made on atomically flat crystalline surfaces. Yet, many surfaces of technological interest are neither crystalline nor atomically flat and this presents a challenge for the assessment of measurement resolution and the ultimate determination of the structures of interest. Problems of friction and adhesion serve as examples in which roughness is a determining factor, and a full understanding of the microscopic interactions requires adequately resolved measurements [1–2].

SiO_2_ grown as a gate dielectric on Si wafers, for example, is amorphous and exhibits stochastic surface roughness. Precise measurement of this roughness by AFM has generated controversy following the widespread use of SiO_2_ as a support for exfoliated graphene, which may be probed with UHV scanning tunneling microscopy (yielding full atomic resolution, as demonstrated by several groups) [3–7]. The controversy arises when STM measurements of graphene/SiO_2_ are compared with AFM measurements of the bare SiO_2_ substrate, because AFM measurements of SiO_2_ generally show a much smoother topography than is shown by STM of graphene/SiO_2_. Motivated by the experimental difficulty in measuring SiO_2_ surfaces, we propose a model to gain insight into this issue.

Here we present experimental findings on SiO_2_ that have motivated the modeling of tip–surface interactions for the case of a corrugated surface. We discuss the issues that arise when the surface is corrugated on relatively small length scales (our best measurements on SiO_2_ yield a correlation length of 8–10 nm). We develop a continuum model that explicitly accounts for a quasi-1-D substrate corrugation (modeled as a sinusoid) and obtain the response of a spherical tip to van der Waals (vdW) interactions. To our knowledge, it is the first model to directly incorporate the lateral variation of van der Waals forces due to surface corrugation and to attempt to quantify this in terms of contours of constant frequency shift. We discuss the first results of this model, specifically showing attenuation of the substrate corrugation in imaging. We also report a deviation from the generally assumed Hamaker force law for the interaction of a sphere with a flat surface (*F* ~ *A_H_R*/6*z*^2^).

## SiO_2_ resolution controversy

Graphene was brought to prominence by the pioneering work of Geim and Novoselov in developing a fabrication technique for graphene devices involving optical identification of exfoliated flakes on 300 nm thick SiO_2_/Si [8]. As a result, much of the early scanning probe investigations were performed on SiO_2_ and questions about the relationship between graphene device properties and substrate properties, including topography, remain prominent in the field of graphene research. The ﬁrst investigations of SiO_2_-supported graphene by means of scanning-probe methods appeared in 2007 [5–6]. These early investigations attributed the roughness of the graphene to the roughness of the underlying SiO_2_. Previously, measurements of suspended graphene by TEM in diffraction mode suggested an “intrinsic” rippling in the graphene structure [9], which presumably originates from the same physics that describes the crumpling of soft membranes [10]. More recently, a study comparing scanning-probe measurements of the corrugation of single-layer graphene (by UHV STM) with that of SiO_2_ (by ambient AFM) reported a signiﬁcantly greater corrugation for the graphene than that observed for the SiO_2_ [4]. These measurements were interpreted as an “intrinsic” rippling of the partially suspended graphene, presumably of the same origin as that observed by TEM for fully suspended graphene [9]. However, any signiﬁcant “suspension” and intrinsic rippling of the graphene over SiO_2_ is hard to reconcile with the energetics of substrate adhesion [11–13].

Our previous work [14] addressed the issue of intrinsic rippling in SiO_2_-supported graphene by presenting high-resolution UHV NC-AFM measurements of the SiO_2_, in which it was shown that there were more small-scale features present on the SiO_2_ than previously measured. The corrugation of bare SiO_2_ was shown to be slightly greater than the corrugation of the graphene over all relevant length scales and, thus, the graphene conforms to the substrate, consistent with the energetics of bending and adhesion. This study helped to resolve questions about the relationship between the substrate and the graphene topography for SiO_2_. Specifically, the higher-resolution measurement of the substrate roughness allowed a quantitative analysis based on theories of membrane adhesion. It also brought to the fore the experimental difficulty of obtaining high-resolution AFM images on corrugated surfaces, given that many previous measurements of SiO_2_ appear to be under-resolved. It is likely that further high-resolution SPM studies will provide breakthroughs in problems that are currently poorly understood, such as the unusually high adhesion energy of graphene to SiO_2_ [15], and its anomalous frictional behavior [16]. Beyond graphene, the use of SiO_2_ is commonplace as a substrate in electronic-device research (carbon-nanotube devices, organic electronics, etc.).

**Figure 1:**
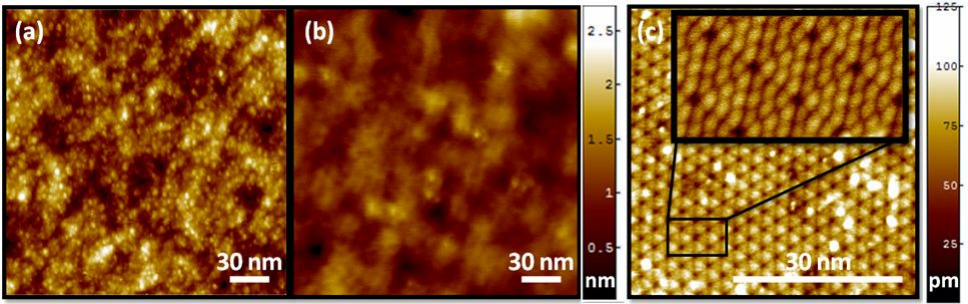
AFM resolution examples: (a) high resolution UHV NC-AFM image of SiO_2_ displaying features with radius of curvature ~2.3 nm (*R*_tip_ nominally 2 nm, ∆*f* = −20 Hz, *A* = 5.0 nm, image size = 200 nm × 200 nm) (b) under-resolved UHV NC-AFM image of SiO_2_ with the same height scale as (a) (*R*_tip_ nominally 30 nm, ∆*f* = −150 Hz, *A* = 1.0 nm, image size 200 nm × 200 nm) (c) UHV NC-AFM image of Si(111) with inset showing atomic resolution (*R*_tip_ nominally 7 nm, ∆*f* = −40 Hz, *A* = 7.1 nm, image size 50 nm × 50 nm).

While one may readily obtain atomic resolution on certain flat surfaces, such as the well-studied (7 × 7) reconstruction of Si(111), obtaining this same level of resolution on rough surfaces presents an experimental challenge. Under suitable conditions, atomic resolution of amorphous surfaces has been achieved. For atomically resolved images of barium silicate glass, UHV contact-mode AFM with a relatively high loading force (25–50 nN) was utilized [17]. Quartz glass has also been measured with comparable resolution, leading to real-space images of the amorphous atomic structure [18]. Despite the atomic resolution obtained for quartz in [18], those measurements fail to account for the observed topography of SiO_2_-supported graphene, due to apparent differences in surface structure between the carefully UHV-prepared quartz in that study and the SiO_2_ substrates used for graphene. As with the barium silicate measurements, for high-resolution measurements of SiO_2_, special conditions were necessary [18]. In order to obtain the high-resolution measurements of the SiO_2_ presented in this paper, a supersharp tip, with a nominal radius of curvature of 2–5 nm, was crucial. Comparing the images obtained with these supersharp tips to those obtained with a metal-coated tip of nominal radius 30 nm, demonstrates the distinct improvement in resolution (Figure 1a and Figure 1b). Features with radius of curvature as small as 2.3 nm were observed in images with the supersharp tip (Figure 1a) [19]. Yet, under comparable experimental conditions, the (7 × 7) structure of Si(111) could be discerned with atomic resolution without the aid of a supersharp tip (Figure 1c). Atomic resolution on Si(111) depends on the short-range chemical forces and the bonding configuration of the tip apex atom [20–23], whereas long-range vdW interactions are a constant background force for AFM imaging of this and other flat surfaces. In contrast, for corrugated surfaces, the vdW interactions will vary laterally and thus play a greater role in determining the contour followed by the probe tip. These experimental observations highlight the difficulty in obtaining adequately resolved NC-AFM measurements on rough, amorphous surfaces and challenge the assumption that, for a given tip radius, the resolution on a rough surface will be comparable to the resolution on a flat surface. While it is the controversy over the resolution of the SiO_2_ substrate that motivates our modeling of AFM resolution for corrugated surfaces, the vdW interaction model itself is more generally applicable to other corrugated surfaces.

## Model of the corrugated-surface resolution

Here we briefly outline the analytic development of the model. Ultimately we wish to find the dependencies of the potential, force, frequency shift, etc., for the case of a spherical tip and a quasi-one-dimensional corrugated surface. The following sections develop the calculation on the assumption that interactions are pairwise additive, beginning with a Lennard-Jones interaction between two atoms [24]. The formalism here closely follows that of [11], in which a detailed analytical theory was developed to model the adhesion of graphene to a sinusoidally corrugated substrate.

This section is presented as follows:

1. Development of the basic formalism for carrying out numerical integration of a Lennard-Jones potential, for a “point atom” interacting with a semi-infinite substrate. By obtaining this “point atom” potential, one can then integrate over the tip volume to obtain the tip–surface potential. We first obtain results for a flat surface with boundary at *z* = 0, initially for the “point atom” and then for a spherical tip body. This allows a check of the numerical integration scheme by comparison with analytical results.
2. We then apply the method to a corrugated surface. As an intermediate result, we discuss the tip–surface potential and its *z* dependence since we find a different scaling from the sphere–plane result generally assumed.
3. Finally, to simulate NC-AFM imaging, we compute frequency shifts for the spherical-tip/corrugated-surface system.

We begin with the Lennard-Jones potential written as

[1]
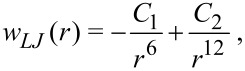


which represents the interaction between a pair of atoms separated by a distance *r*. Following the Hamaker procedure, we assume that the total interaction energy (atom–surface or tip–surface) is obtained pairwise by integration of this potential.

### 1 Atom–surface potential

We first consider a “point atom” interacting with a flat, semi-infinite substrate with density ρ*_S_* (number/volume). The integration may be written as

[2]
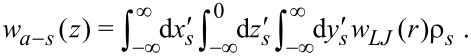


As shown in [25], this has an analytic solution. For a general potential described by

[3]
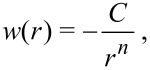


the substrate-integrated potential is

[4]
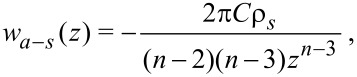


and this is valid for *n* > 3. Here *z* represents the distance from the “point atom” to the substrate surface. We use subsript “*a*–*s*” to denote that this is a potential for an “atom” interacting with the semi-infinite substrate.

For *n* = 6 (the usual attractive vdW form) this reduces to

[5]
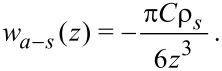


Combining the attractive *r*^−6^ term and repulsive *r*^−12^ term, the result may be expressed as

[6]
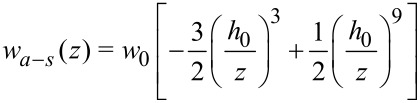


with

[7]
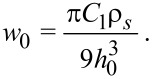


**Figure 2:**
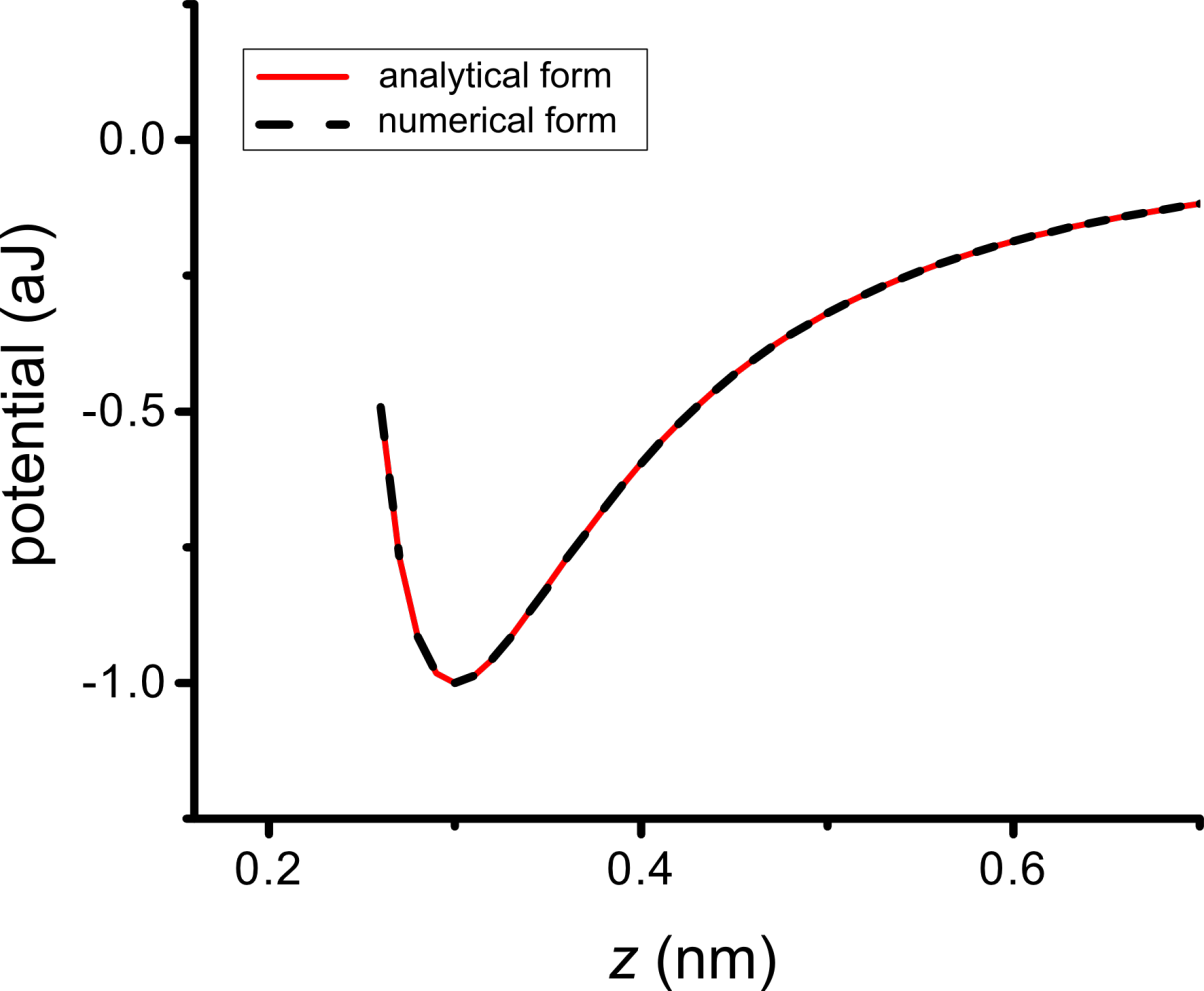
Verification of atom–substrate potential: Potential *w_a_*_–_*_s_* versus *z* for numerical and analytical schemes for a “point atom” interacting with a flat surface. The near-perfect overlap of the curves demonstrates the fidelity of the numerical integration scheme.

By inspection, it is apparent that Equation 6 represents a potential with depth *w*_0_ at distance *h*_0_ from the surface. Additionally, one sees that choosing (*w*_0_, *h*_0_) is equivalent to choosing (*C*_1_, ρ*_s_*), according to Equation 7. Thus, in our numerical implementation we choose values for *w*_0_ and *h*_0_. As a first check on our substrate by numerical integration, we compare the numerical integration of Equation 2 with the analytical result in Equation 6. In this case the interaction is parameterized as *w*_0_ = 1.0 aJ and *h*_0_ = 0.3 nm. The agreement is excellent, as shown in Figure 2.

### 2 Tip–surface potential

Once the atom–surface potential is obtained, the tip–surface potential is obtained in an analogous manner. It is computed as

**Figure 3:**
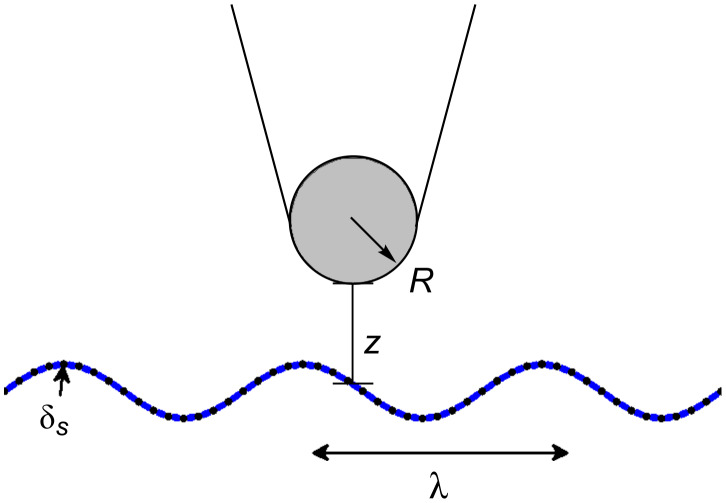
Schematic illustrating the model geometry: The surface is sinusoidally corrugated along the *x* direction only, with wavelength λ and amplitude δ*_s_.* The surface corrugation is independent of *y* (quasi-1-D geometry). The tip is modeled as a sphere of radius *R*.

[8]
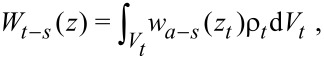


where the uppercase *W* designates a potential between two extended objects. Here, ρ*_t_* is the tip density (number/volume) and the integration is over the (spherical) tip volume. The *z* coordinate for *W_t_*_–_*_s_*(*z*) is the distance between the surface and the apex of the spherical tip (the point closest to surface), as depicted in Figure 3. Employing the NIntegrate function in Mathematica 8.0, the numerical integration of Equation 2 generates the atom–surface potential as a tabulated function of *z*, with scaling determined by (*w*_0_, *h*_0_). We then numerically integrate this tabulated function over the spherical tip volume, for varying tip–surface distance *z*, using an approach that incorporates the IDL routines INTERPOLATE and INT_3D. As a check on this numerical integration, we compare against the exact analytical result for a sphere attracted to a flat surface by van der Waals forces. It is well-known that the sphere–plane Hamaker integration has the approximate solution [25]:

[9]
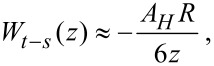


in the limit *z* << *R*, where *A_H_* is the Hamaker constant for the tip–surface material system, given by *A_H_* = *C*_1_·π^2^·ρ*_s_·*ρ*_t_*. Equation 9 is sometimes used for fitting the vdW background in NC-AFM experiments [26–27]. However, for the tip radii modeled here, the limiting approximation is not accurate enough to serve as a test for the tip integration scheme, and we use the following exact analytical expression:

[10]
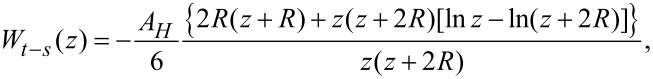


**Figure 4:**
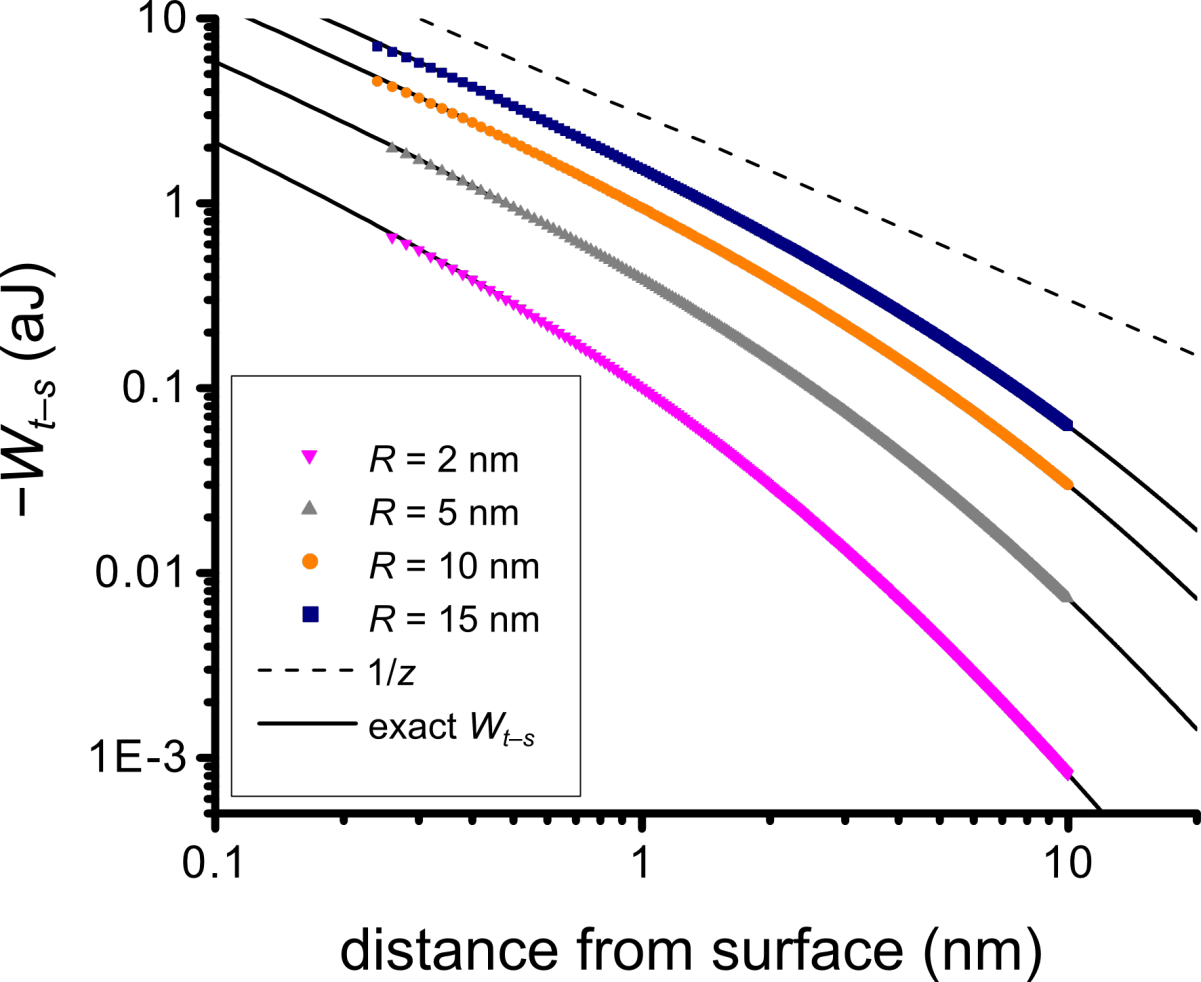
Hamaker force for flat surfaces: Relationship between tip potential and distance from the surface. Here the distance is taken relative to the surface position (distance from surface = *z*(*x*) − *z*_s_(*x*)). The dashed line is a reference for the 1/*z* dependence expected from the Hamaker force law for the interaction between a flat surface and a sphere. The numerical results show excellent agreement with the exact potential (Equation 10).

In Figure 4, we plot −*W_t–s_* versus *z* to show that the numerical integration over the tip volume accurately reproduces the exact formula. Additionally, we plot the function *z*^−1^ to indicate the small-*z* limiting behavior. In all numerical calculations the full Lennard-Jones potential of Equation 6, including both the attractive and the repulsive terms, is utilized. While the analytical expression in Equation 10 is limited to the attractive interaction, the agreement in Figure 4 is excellent.

Following these consistency checks on the numerical integrations with a flat substrate surface as a reference, we now extend the calculation to a corrugated surface. The treatment follows that of [11]; in analogy with Equation 2 the atom–substrate potential is written as

[11]
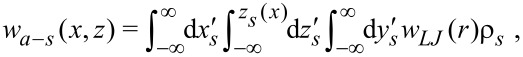


with the essential difference being the upper integration limit on *z*. The upper integration limit in *z* is now the (sinusoidal) surface profile *z_s_*(*x*), given by *z_s_*(*x*) = δ*_s_*·sin (2 π*x*/λ). Note that *w_a_*_–_*_s_* is necessarily a function of *x* and *z*. The tip–surface potential is obtained in analogy with the calculation for the flat surface (Equation 8), and is also a function of *x* and *z*.

All computations are carried out with λ = 10 nm, δ*_s_* = 0.5 nm, *w*_0_ = 0.169 aJ, and *h*_0_ = 0.3 nm. The particular choice of amplitude and wavelength is based on our best measurements of SiO_2_, which gave rms roughness ~0.38 nm and correlation length ~10 nm. The 10 nm period is divided into 16 intervals at which points the potential is calculated (shown as black dots on the sinusoidal surface in Figure 3). In the *z* direction, the grid is much finer, namely 0 to 40 nm in increments of 0.01 nm. The 40 nm range is necessary to incorporate realistic tip diameters, and to allow proper integration over the oscillation amplitude, as discussed below. Our scheme is motivated by simplicity; however, an adaptive grid scheme would be desirable to deal with the rapidly varying behavior of *w_a–s_* near the surface and very smooth asymptotic behavior several nm from the surface.

The computation of the atom–surface potential *w_a–s_*(*x*,*z*) for the corrugated surface requires some careful discussion. In [11], analytical formulas were derived for the integration given in Equation 2. However, the formulae developed there ultimately make the approximation that z >> δ*_s_*, and consequently they do not work well at relatively small *z* (anomalies begin to appear even >1 nm from the surface contour). This is why a final numerical integration was adopted in our work to obtain *w_a–s_*(*x*,*z*). There appear to be inherent numerical difficulties in computing the integral for a sinusoidal surface, and we are currently limited in the closest distance to the surface for which we can compute *w_a–s_*. For example, in the case of the flat substrate, our numerical integration routine allows computation of *w_a–s_* to within 0.19 nm of the surface. In that case, the potential is in the highly repulsive regime with a value of about +24.60*w*_0_, where *w*_0_ is the depth of the potential well at the minimum. The equivalent calculation for a corrugated surface with δ*_s_* = 0.5 nm and λ = 10 nm is generally limited to ~0.26 nm throughout most of the corrugation period (the potential cannot be computed closer than 0.26 nm to the surface). The limits on *w_a–s_* carry over directly into limits on *W_t–s_*, as we only integrate the tip potential where the integrand is defined. Thus within our continuum model with a perfectly rigid tip and substrate, we cannot generally take the tip into the regime in which the *overall* interaction is repulsive. This is rather unsatisfactory at present, as it would be preferable to have well-defined numerical values (even if unrealistically large), and then let the limits of the model be decided on physical grounds, i.e., peak force or stress on the tip apex, etc.

### 3 Calculation of frequency shifts

Once the tip–surface interaction potential *W_t–s_* is obtained, the interaction force *F_t–s_* is found straightforwardly by differentiation with respect to *z*. We then compute the frequency shift using the following expression [28], which is exact to 1st order in classical perturbation theory:

[12]
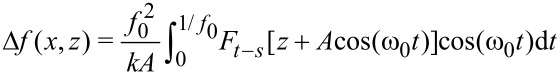


with spring constant *k* = 40 N/m and resonant frequency *f*_0_ = 300 kHz. We then convert to the normalized frequency shift γ, which is defined as [20]

[13]
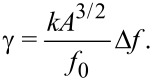


## Results and Discussion

Using the model, we arrive at several key results. First, we find that the generally assumed Hamaker force law for the interaction between a spherical tip and a flat surface does not hold in the case of corrugated surfaces. Second, we find that the imaged structure is attenuated with respect to the surface geometry, even for small distances between the tip and the sample.

### Deviation from the sphere–plane Hamaker force law

In the previous section, we discussed the Hamaker integration for a sphere interacting with a flat surface through van der Waals forces. The integration can be carried out without approximation to yield the exact formula; this exact formula is cumbersome and given by Equation 10. In the limit *z* << *R*, this formula simplifies greatly to *W_t–s_* ≈ −*A_H_R*/6*z*, which is often used in describing tip–sample vdW forces. Applying the formalism developed for a sinusoidally corrugated surface, we find that the basic scaling with distance is fundamentally different when the surface is corrugated.

**Figure 5:**
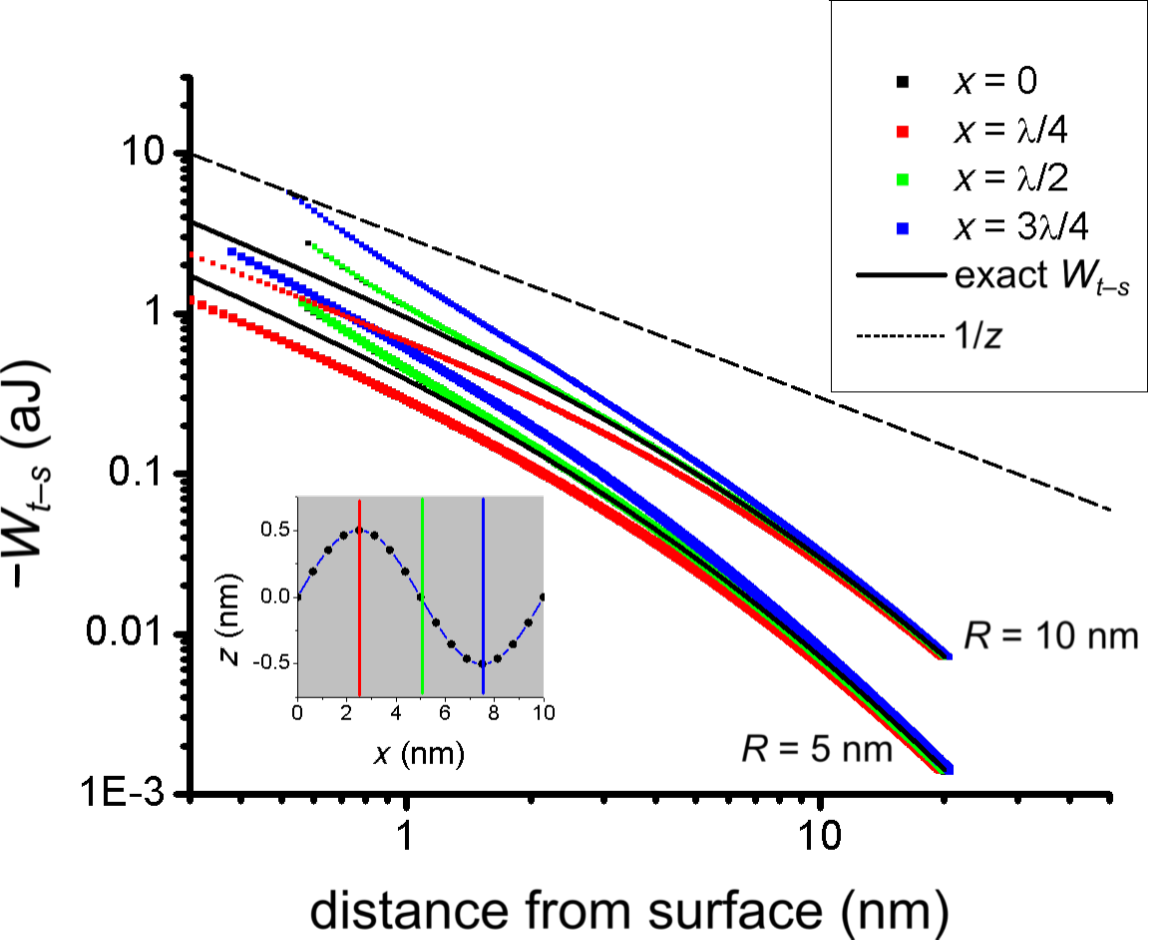
Hamaker force law for corrugated surfaces: Tip–sample distance dependence of tip potential for high-symmetry points (inset, *x* = 0, *x* = λ/4, *x* = λ/2, and *x* = 3 λ/4) for the two radii (*R* = 5 nm and *R* = 10 nm); for *x* = 0 and *x* = λ/2 the curves overlap. Lines for the exact analytical form (Equation 10) of the Hamaker relationship between a sphere and a plane are shown (black line) for comparison.

Figure 5 shows the relationship between *W_t–s_* and the local height above the surface (*h* = *z*(*x*) − *z_s_*(*x*)) for tip radii of 5 nm and 10 nm, at four high symmetry points on the corrugated surface (*x* = 0, *x* = λ/4, *x*= λ/2, and *x* = 3λ/4). We compare the curves derived from the corrugation model to the exact curves corresponding to a flat surface, and additionally show the reference curve 1/*z*, which represents the small-*z* limiting behavior for the flat surface. We see that, unlike the flat case, the curves do not show a 1/*z* dependence in the limit of small tip–sample distances. Assuming a relationship of the form 1/*z*^β^ for *W_t–s_* versus the tip–sample distance, we find β > 1. This means that the tip potential drops off more quickly with increasing distance than one would expect from application of the Hamaker force law for the relationship between a sphere and a plane. Additionally, the dependence of *V*_tip_ on the tip–sample distance varies with lateral position, showing the strongest distance dependence at the valley position (*x* = 3λ/4, blue curve) and the weakest distance dependence at the peak position (*x* = λ/4, red curve). For *x* = 0 and *x* = λ/2, the distance dependencies are equivalent, which is consistent with the observation that these two locations are mirror symmetric in geometry. For all lateral positions studied, a departure from the sphere–plane Hamaker force law results. The departure is most pronounced when the tip is in close proximity to the surface; as the distance from the surface increases the potential converges to the exact result for a flat surface. While the deviation from the sphere–plane Hamaker force law is not mapped throughout the corrugation (λ, δ) parameter space here, we expect that for a given tip radius the deviation will decrease with longer λ and smaller δ due to decreased interaction between the tip and the substrate side walls. This prediction is consistent with the flat surface case, which is restored in the limits λ → ∞ and δ → 0.

### Attenuation of surface features

**Figure 6:**
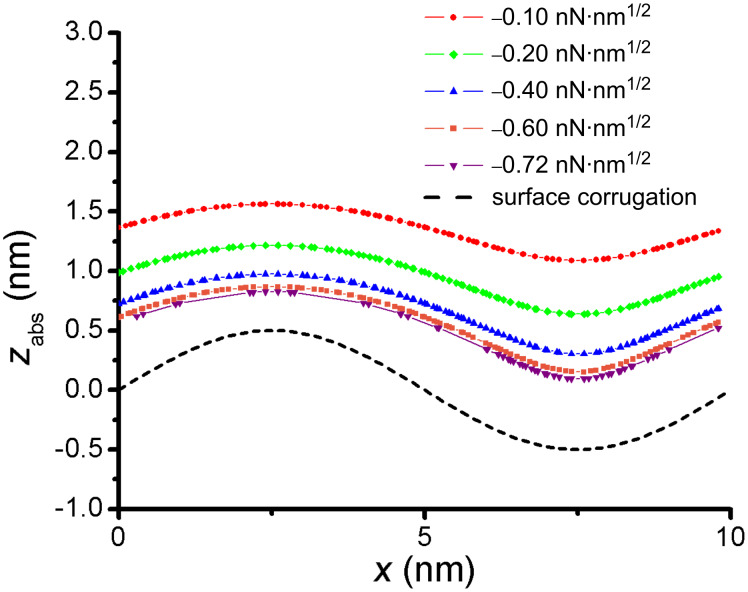
Contours of constant normalized frequency shift, γ, for a corrugated surface. Attenuation is observed as the distance from the surface increases. Here, *z*_abs_ gives an absolute position in the *z* direction, not a relative distance from the surface.

To determine the degree of attenuation of surface features for NC-AFM, contours of constant frequency shift were calculated (Figure 6) by using the method described in the previous section. Here, we present results for a tip with radius 5 nm. With increasing distance from the surface these contours show attenuation of the corrugation. As discussed in the previous section, the proximity to the surface is limited by our first numerical integration to obtain *w_a–s_*. At our computational limit, the nearest contour that we can calculate corresponds to a normalized frequency shift of −0.72 nN·nm^1/2^ (−22.8 fN·m^1/2^), which is well into the range in which atomic-resolution images are normally obtained [20]. Most significantly, at this interaction level the contours are attenuated by ~30% (lower-most contour, purple curve in Figure 6). At −0.1 nN·nm^1/2^ (upper-most contour, red curve in Figure 6) the model predicts over 50% attenuation compared to the surface corrugation. The attenuation of surface features can be understood intuitively by considering the vdW interaction of the tip and the corrugated sample surface. For flat surfaces, the vdW interaction provides a constant background and is most strongly concentrated at the tip apex, but for corrugated surfaces the vdW interactions over peak positions and valley positions are different and interactions with the side of the tip become more important. For the valley positions, the attractive force between the tip and the sides of the valley will lead to a stronger attraction than for the flat surface case and thus result in a higher *z* position for the same frequency shift. A similar physical argument can be made for the peak positions. In this case the downward slope means that neighboring atoms are farther away, the vdW interactions with these atoms is smaller compared with the flat case due to the increased distance, and as a result the same frequency shift will occur at a lower *z* position. The vdW interactions with neighboring atoms become more dominant at larger *z* distances (smaller frequency shifts), and therefore one can intuitively expect greater attenuation (lowering of peak positions and heightening of valley positions) based on these simple, physical vdW arguments. A similar argument was presented by Sun et. al in describing the attenuation in the graphene moiré structure on Ir(111) due to the vdW interaction between the tip and the underlying Ir(111) structure [29]. While attenuation is to be expected for increased distance between the tip and the sample, we emphasize that the degree of attenuation for a tip of 5 nm radius is significant even at small distances, with a normalized frequency shift that is relatively large. To obtain accurate experimental results with NC-AFM it is of critical importance to choose a frequency shift setpoint such that the distance between the tip and the surface is minimized, *especially* when seeking accurate topography of corrugated surfaces. The model used does not account for local bonding, electrostatic forces, or atomistic interactions beyond the inclusion of a pairwise vdW interaction, all of which affect the AFM resolution; nonetheless, even if these interactions were included, the varying vdW and resultant attenuation of features still presents a problem to resolution.

## Conclusion

As is already well known in the field of atomic force microscopy, a sharp tip and close proximity to the surface is the key to obtaining accurate topographic images with high resolution. Here we have shown that, even more so than for flat surfaces, these factors are especially important for high-resolution imaging of rough surfaces, based only on the differences between vdW interactions. While the model results support the experimental difficulty of obtaining accurate images of rough surfaces, the model itself oversimplifies the multifaceted complexities of experimental AFM setups. More complex models, which include short-range bonding and electrostatic forces, more realistic tip geometries, and calculations for closer proximities between tip and sample, are needed for a more complete and quantitatively accurate understanding of the factors limiting the resolution of corrugated surfaces.

## Experimental

All NC-AFM images were collected with a JEOL ultrahigh-vacuum atomic force microscope with a base pressure of 4 × 10^−8^ Pa. SiO_2_ samples (Figure 1a and Figure 1b) were cleaved in air to the proper size then quickly transferred into the ultrahigh-vacuum JEOL AFM system (4500A, Nanonis controller). SiO_2_ samples were baked at 130 °C for cleaning. In order to replicate the experimental substrate preparation often used for graphene exfoliation, no additional cleaning procedures were performed before imaging of the SiO_2_. Si(111) samples (Figure 1c) were cleaned in UHV by the standard procedure with repeated flashing at 1530 K, followed by slow cooling through the (1 × 1)-to-(7 × 7) phase transition [30]. NC-AFM measurements were performed with commercially available cantilevers; supersharp tips were used for the high-resolution SiO_2_ measurements (Veeco TESP-SS with nominal radius of 2–5 nm), metal-coated Si for the under-resolved SiO_2_ measurements (MikroMasch DPER15), and uncoated Si for the Si(111) measurements (Nanosensors Point Probe NCH with nominal radius 7 nm). All images are presented in raw form, with only a plane-fit background subtraction. Commercial software (SPIP) was used for the presentation of image data.

## References

[1] Luan B, Robbins M O (2006). Phys Rev E.

[2] Zappone B, Rosenberg K J, Israelachvili J (2007). Tribol Lett.

[3] Deshpande A, Bao W, Miao F, Lau C N, LeRoy B J (2009). Phys Rev B.

[4] Geringer V, Liebmann M, Echtermeyer T, Runte S, Schmidt M, Rückamp R, Lemme M C, Morgenstern M (2009). Phys Rev Lett.

[5] Ishigami M, Chen J H, Cullen W G, Fuhrer M S, Williams E D (2007). Nano Lett.

[6] Stolyarova E, Rim K T, Ryu S, Maultzsch J, Kim P, Brus L E, Heinz T F, Hybertsen M S, Flynn G W (2007). Proc Natl Acad Sci U S A.

[7] Zhang Y, Brar V W, Girit C, Zettl A, Crommie M F (2009). Nat Phys.

[8] Novoselov K S, Geim A K, Morozov S V, Jiang D, Zhang Y, Dubonos S V, Grigorieva I V, Firsov A A (2004). Science.

[9] Meyer J C, Geim A K, Katsnelson M I, Novoselov K S, Booth T J, Roth S (2007). Nature.

[10] Nelson D R, Peliti L J (1987). J Phys (Paris).

[11] Aitken Z H, Huang R (2010). J Appl Phys.

[12] Li T, Zhang Z (2010). J Phys D: Appl Phys.

[13] Pierre-Louis O (2008). Phys Rev E.

[14] Cullen W G, Yamamoto M, Burson K M, Chen J H, Jang C, Li L, Fuhrer M S, Williams E D (2010). Phys Rev Lett.

[15] Koenig S P, Boddeti N G, Dunn M L, Bunch J S (2011). Nat Nanotechnol.

[16] Lee C, Li Q, Kalb W, Liu X-Z, Berger H, Carpick R W, Hone J (2010). Science.

[17] Raberg W, Wandelt K (1998). Appl Phys A: Mater Sci Process.

[18] Raberg W, Ostadrahimi A H, Kayser T, Wandelt K (2005). J Non-Cryst Solids.

[19] Burson K M, Yamamoto M, Cullen W G (2012). High resolution microscopy of SiO2 and the structure of SiO2-supported graphene. Proceedings of the ASME 2011 International Design Engineering Technical Conferences & Computers and Information in Engineering Conference IDETC/CIE 2011.

[20] Giessibl F J (2003). Rev Mod Phys.

[21] Lantz M A, Hug H J, van Schendel P J A, Hoffmann R, Martin S, Baratoff A, Abdurixit A, Güntherodt H-J, Gerber C (2000). Phys Rev Lett.

[22] Minobe T, Uchihashi T, Tsukamoto T, Shigeki O, Sugawara Y, Morita S (1999). Appl Surf Sci.

[23] Pérez R, Štich I, Payne M C, Terakura K (1998). Phys Rev B.

[24] Hölscher H, Allers W, Schwarz U D, Schwarz A, Wiesendanger R (2000). Phys Rev B.

[25] Israelachvili J N (2011). Intermolecular And Surface Forces.

[26] Hölscher H, Schwarz A, Allers W, Schwarz U D, Wiesendanger R (2000). Phys Rev B.

[27] Lantz M A, Hug H J, Hoffmann R, van Schendel P J A, Kappenberger P, Martin S, Baratoff A, Güntherodt H-J (2001). Science.

[28] Giessibl F J (2001). Appl Phys Lett.

[29] Sun Z, Hämäläinen S K, Sainio J, Lahtinen J, Vanmaekelbergh D, Liljeroth P (2011). Phys Rev B.

[30] Conrad B R, Cullen W G, Riddick B C, Williams E D (2009). Surf Sci.

